# Applying neural ordinary differential equations for analysis of hormone dynamics in Trier Social Stress Tests

**DOI:** 10.3389/fgene.2024.1375468

**Published:** 2024-08-01

**Authors:** Christopher Parker, Erik Nelson, Tongli Zhang

**Affiliations:** ^1^ Department of Pharmacology and Systems Physiology, University of Cincinnati College of Medicine, Cincinnati, OH, United States; ^2^ Department of Psychiatry and Behavioral Neuroscience, University of Cincinnati College of Medicine, Cincinnati, OH, United States

**Keywords:** major depressive disorder (MDD), machine learning (ML), artificial intelligence (AI), neural network, dynamical system

## Abstract

**Introduction:** This study explores using Neural Ordinary Differential Equations (NODEs) to analyze hormone dynamics in the hypothalamicpituitary-adrenal (HPA) axis during Trier Social Stress Tests (TSST) to classify patients with Major Depressive Disorder (MDD).

**Methods:** Data from TSST were used, measuring plasma ACTH and cortisol concentrations. NODE models replicated hormone changes without prior knowledge of the stressor. The derived vector fields from NODEs were input into a Convolutional Neural Network (CNN) for patient classification, validated through cross-validation (CV) procedures.

**Results:** NODE models effectively captured system dynamics, embedding stress effects in the vector fields. The classification procedure yielded promising results, with the 1x1 CV achieving an AUROC score that correctly identified 83% of Atypical MDD patients and 53% of healthy controls. The 2x2 CV produced similar outcomes, supporting model robustness.

**Discussion:** Our results demonstrate the potential of combining NODEs and CNNs to classify patients based on disease state, providing a preliminary step towards further research using the HPA axis stress response as an objective biomarker for MDD.

## Introduction

The use of Machine Learning (ML) and Artificial Intelligence (AI) for data analysis and pattern recognition has ballooned into a $500+ billion industry over the past several years (Artificial Intelligence Market, 2023), leading to myriad advances in academic disciplines as well. As part of the ML/AI advances, active research is ongoing to implement these methods for diagnostic assistance in clinical settings (Giordano et al., 2021; Huang et al., 2023), for precision medicine (Johnson et al., 2021), and for drug discovery and development (Mak et al., 2023).

This led us to explore the application of Neural Ordinary Differential Equations (NODEs) (Chen et al., 2018) to physiological systems (Lu et al., 2021a; Lu et al., 2021b; Bräm et al., 2023). Inspired by these works, we applied ML/AI to analyze dynamical hormone data from the hypothalamic-pituitary-adrenal (HPA) axis. The HPA axis is the primary regulator of cortisol, a steroid hormone involved in many physiological processes but primarily associated with the stress response. We have specifically examined the response of the HPA axis in healthy controls and subjects with Major Depressive Disorder (MDD) while the patients undergo Trier Social Stress Tests (TSST) (Kirschbaum et al., 1993; Allen et al., 2017).

In the presence of a stressor, the paraventricular nucleus (PVN) of the hypothalamus releases corticotropin-releasing hormone (CRH) into the hypophyseal portal system for transport to the anterior pituitary (Smith and Vale, 2006). Increased CRH concentration causes the anterior pituitary to release adrenocorticotropic hormone (ACTH) into the circulatory system. When circulating ACTH reaches the adrenal glands, it stimulates production of cortisol (Smith and Vale, 2006). ACTH and cortisol are easily measured from blood, while CRH is not released into the systemic circulation and therefore cannot be easily measured.

In this work, we apply NODE models to replicate hormone changes in patients undergoing TSST without prior knowledge of the stressor. Additionally, the trained model can forecast stress effects in new situations. Dynamic analysis indicates that the stress effect is embedded in the non-autonomous vector fields derived from the NODE model. These time-varying vector fields (represented in 3-dimensions) can then be used as input for a subsequent CNN. Our research illustrates how this combined pipeline of NODEs and CNNs can effectively classify patients from our dataset based on disease state. This represents a first step towards clinical applications using the HPA axis stress response as an objective biomarker for MDD. We also address the current limitations of our results and suggest possible improvements.

## Results

**FIGURE 1:**
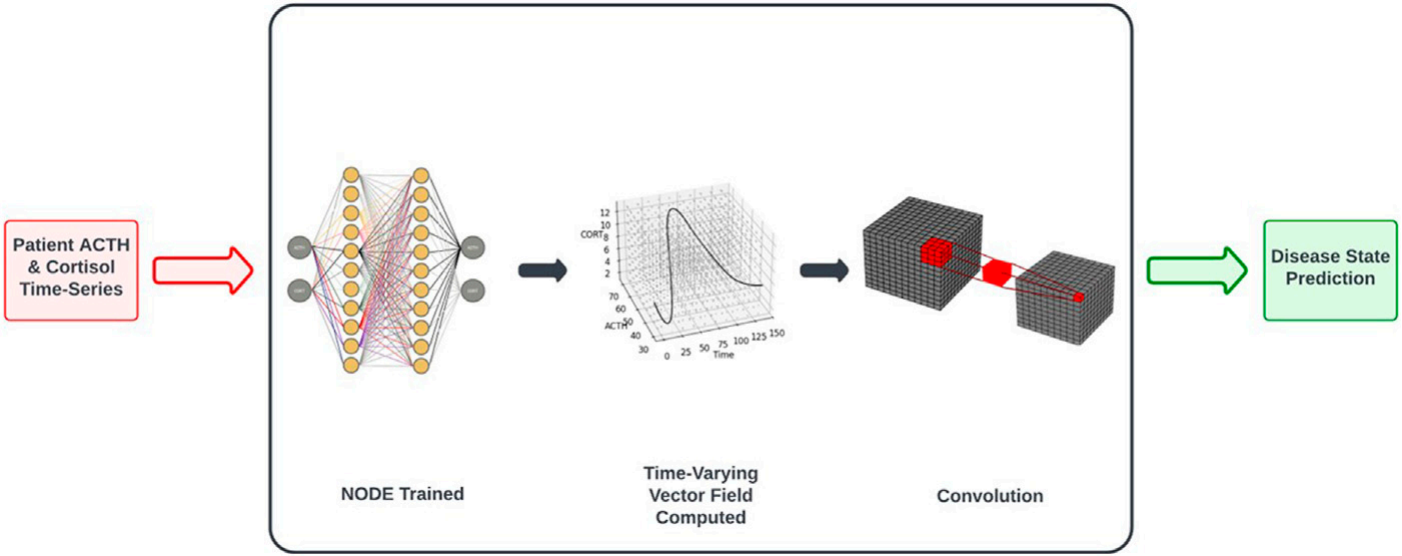
Workflow of control vs. MDD classification based on NODE encodings of the underlying system dynamics. See Classification Based on Trained NODEs subsection of Results for a more complete description of the procedure.

Figure 1 depicts the process by which classification decisions are made. We will discuss each step in turn, starting with the inputs to the system. The procedure requires the full time-series dataset for a patient, along with the time at which each datapoint was collected. We also need to have labels for the data, indicating the correct classification decision (for use when training the CNN in the final step of the process). See the Classification was performed based on trained NODEs subsection of Results for a description of the classification training procedure.

### An augmented NODE system captures system dynamics accurately

The NODE architecture utilizes a feedforward artificial neural network (ANN) as the right-hand side of an ODE, and the ANN encodes a vector field. The NODE system is passed the initial conditions for time, ACTH and cortisol (by adding the time dimension to the inputs, we create an Augmented NODE system, as in Dupont et al. (2019), along with the time interval for integration. Due to the non-autonomous nature of the system being modeled (because stress is input at 30 min for each time-series), this dimension augmentation allows for more accurate learning of system dynamics. The ANN representing the vector field then outputs the instantaneous change in time, ACTH and cortisol. If the initial condition is varied, then the NODE yields different flows through the vector field it represents, equivalent to a traditional ODE system.

We remove time points iteratively from the beginning of an individual time-series dataset, obtaining 10 time-series from the 11 time points contained in the data, and train the NODE on all of these samples. Training the network with various initial time points allows the network to better learn the time dependence of the system, since networks trained without this procedure did not exhibit the same degree of time-dependent variation in the vector fields.

**FIGURE 2:**
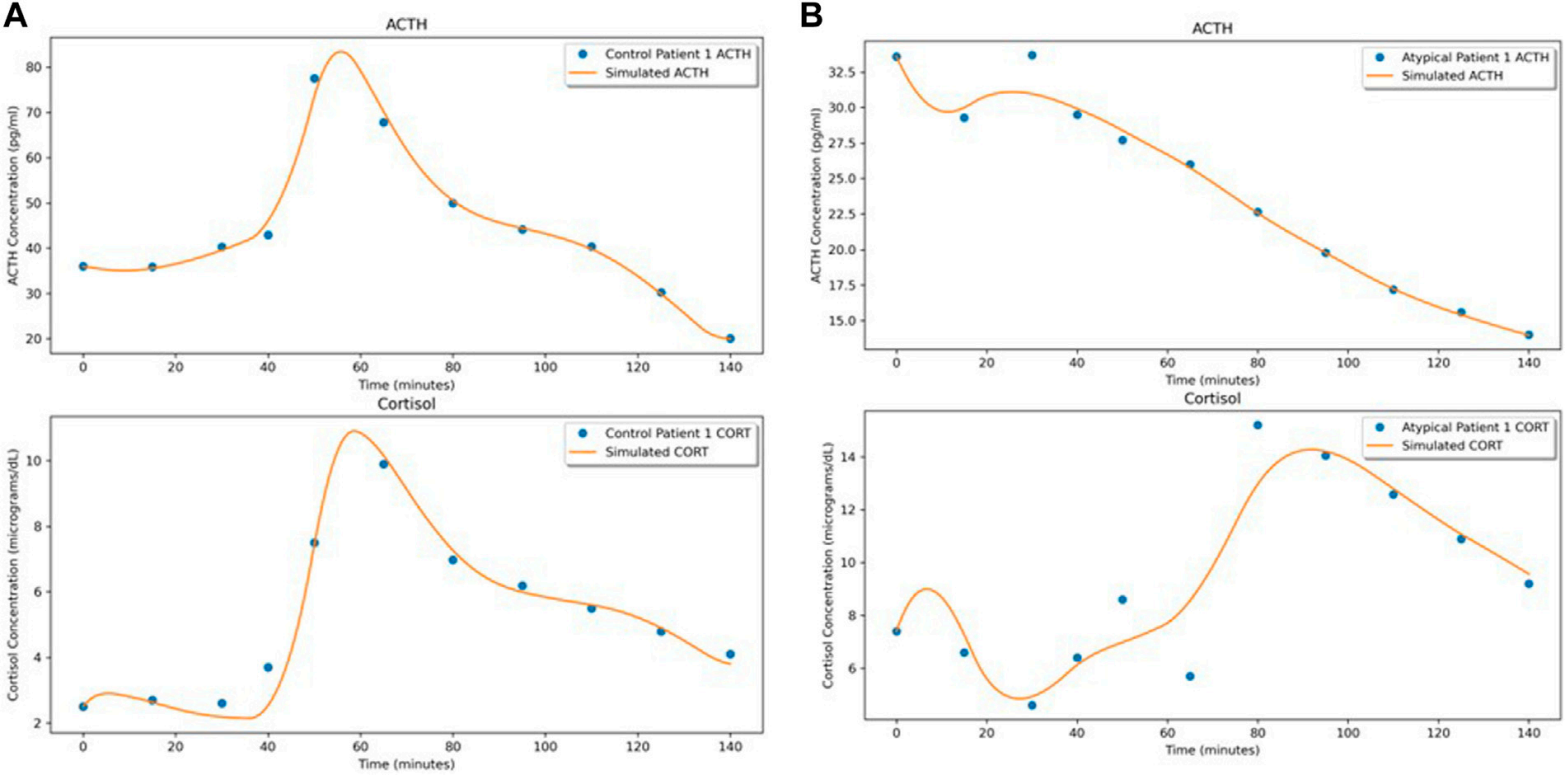
NODE fits for the first subject in **(A)** Control and **(B)** Atypical MDD. In both **(A,B)**, the upper graph shows the ACTH concentration time series and the lower graph shows the cortisol concentration time series.

By solving this system (with the Python package torchdiffeq by Chen et al. (2018) in which the system is largely solved using the standard Python ODE solvers from the scipy package), we obtain time series of predicted values. Using the mean squared error (MSE) loss, we compare the predictions to the dataset for backpropagation. Training is stopped after 2,000 iterations or when the maximum MSE loss (for all 10 time-series) drops below 5% of the overall mean of the full dataset. See Figure 2 for examples of the predictions compared to the individual datasets (depicted are the first subjects from the Control and Atypical groups).

**FIGURE 3:**
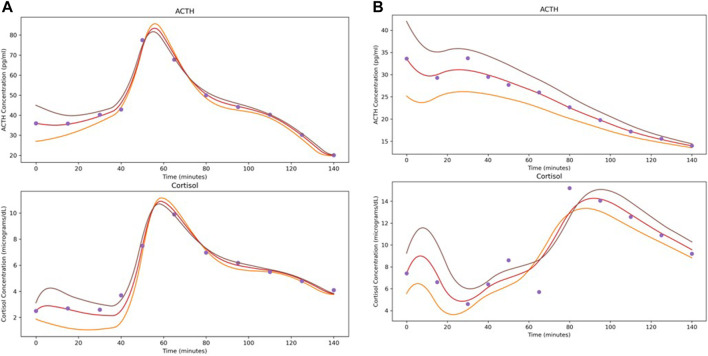
Demonstration of the effects of varying initial conditions by ±25% when solving the system learned by the NODE networks. Depicted subjects are the first subject from **(A)** Control and **(B)** Atypical MDD.

As an illustration of the NODE system as a vector field, Figure 3 shows the predicted time series when the initial conditions for ACTH and cortisol are adjusted by ±25%. The modifications to the initial conditions cause the flow to shift slightly. It is interesting to note that though stress signal is not part of the underlying NODE system, since we do not have data on its level, its effect on the hormone level changes were faithfully recaptured by the NODE encoder.

### The stress effect is embedded in the non-autonomous vector fields computed from the NODE model

**FIGURE 4:**
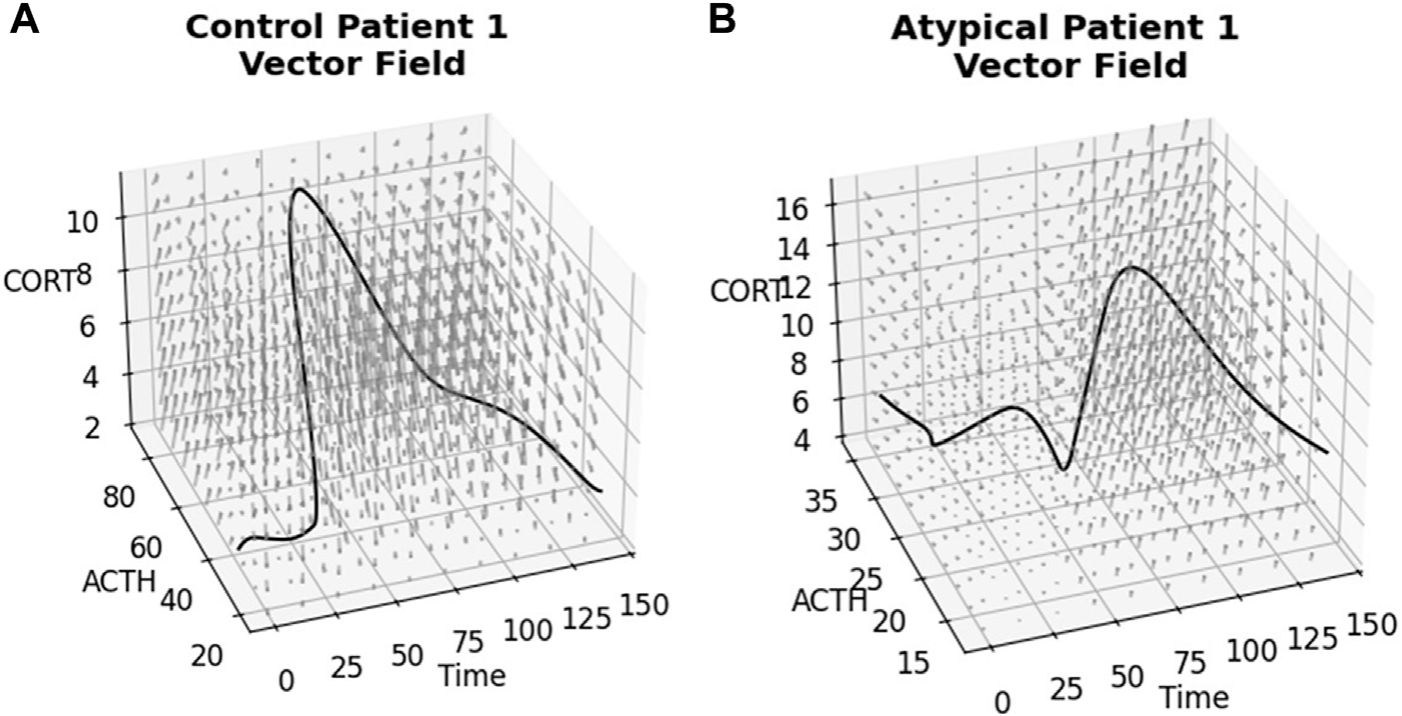
Time-varying vector fields learned from the data for the first subject from **(A)** Control and **(B)** Atypical MDD. Axes are ACTH concentration in pg/mL, cortisol concentration in μg/dL and time in minutes. Rotating.gif versions of these plots are available in the Supplementary Material.

**FIGURE 5:**
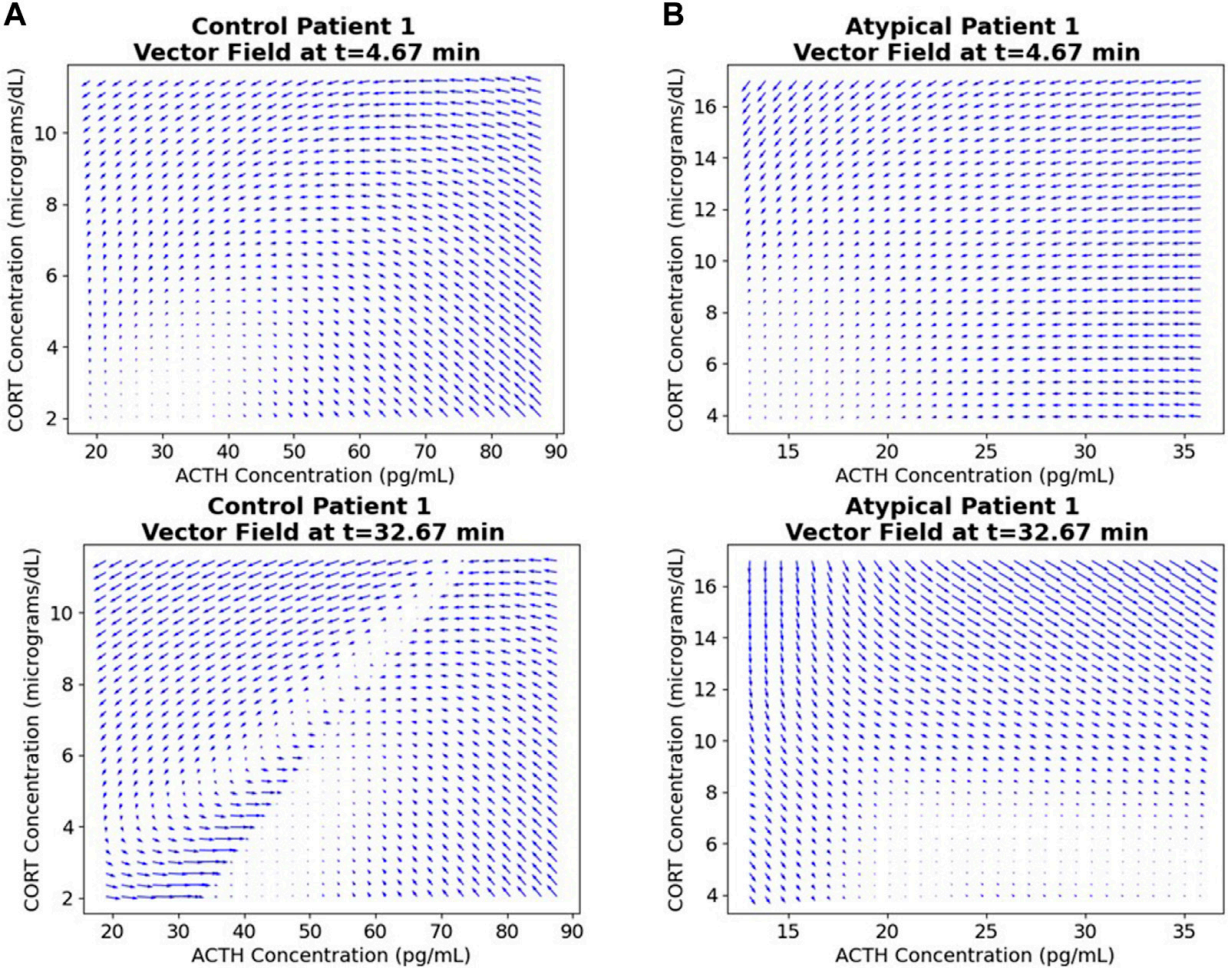
ACTH-cortisol plane at two different times for the first subject from **(A)** Control and **(B)** Atypical MDD. We see changes depending on time, as the network has learned the nonautonomous system dynamics.

The ANN resulting from the above NODE training procedure is used to obtain a time-varying vector field by passing triples of (time, ACTH, cortisol) and obtaining a vector representing change in these values at that point. See Figure 4 for examples of these 3-dimensional vector fields learned from the individual subject time-series data. The subjects correspond to those shown in Figures 2, 3 and the flows through the vector fields are consistent with those figures. Figure 5 shows the ACTH-cortisol plane at two different times for the same patients, demonstrating the time-dependence of the system learned from the data.

Due to the time-varying nature of the 3-dimensional vector fields, we can see the effect of the stressor on the ACTH-cortisol plane when the patients’ hormone concentrations respond. The stressor was initiated at 30 min and ceased at 50 min in the TSST procedure. In Control Patient 1 (shown in Figures 2A–4A), the system behaves as we would expect for the observed hormone response to the stressor starting 10–15 min after it is initiated. The effect of the stressor is not apparent until roughly 20–30 min after initiation of the stressor in Atypical MDD Patient 1 (shown in Figures 2B–4B). This is caused by the unusual nature of the stress response in this patient, which the system is still able to effectively learn from the data.

These vector fields contain a considerable amount of information about the stress response of individual subjects, so we used the vector fields as inputs in a classification procedure.

### Classification was performed based on time-varying vector field representations

Next, 10 × 10 × 10 × 3 representations of the vector fields for each individual were extracted and used with a CNN for classification. The representations were created by sampling 10 points for each of time, ACTH and cortisol and the corresponding 3-dimensional vector of their instantaneous change at that point. The samples were linearly spaced between the minimum value in the time series minus 5% and maximum value in the time series plus 5% for each variable.

Due to the limited availability of data for our tests, we were concerned about the possibility of overfitting. By expanding the 11 features of each individual patient to a 3D vector field, we increased the number of features used for classification substantially. To assess the impact of potential overfitting, we performed two separate cross-validation (CV) procedures wherein we tested the classification on many subsets of the data. These CV procedures allowed us to obtain an estimate of generalizability of the classification results. This involved leaving out one sample from each group as test data, training on the vector fields of the remaining individuals and comparing predicted classes to the labels using binary cross-entropy loss. This was repeated for all pairs of one subject from each group, a total of 210 train/test splits (15 control and 14 Atypical MDD subjects) which we term 1 × 1 CV. We also performed a similar procedure leaving out two subjects from each group, which we term 2 × 2 CV (9,555 train/test splits performed).

**FIGURE 6:**
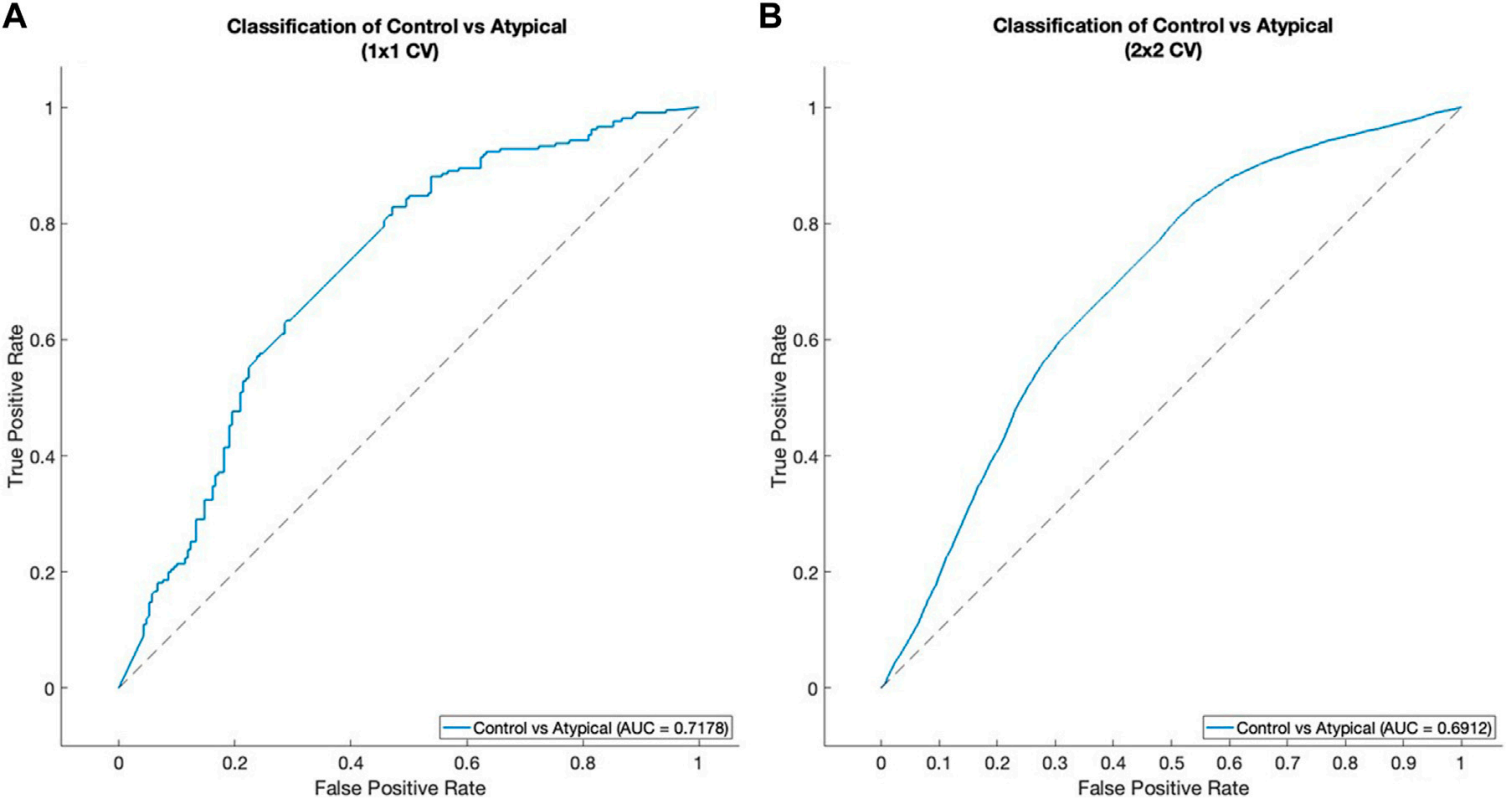
ROC curves for classification between healthy control subjects and Atypical MDD subjects. **(A)** 1 × 1 CV, **(B)** 2 × 2 CV. See Classification was performed based on time-varying vector field representations subsection of Results for details of the cross-validation procedure.

The classification results were evaluated using the area under the receiver operating characteristic curve (AUROC) as a metric (Prusty et al., 2022). Figure 6A shows the result of the 1 × 1 CV procedure, while Figure 6B shows the results for the 2 × 2 CV. Both procedures give roughly the same relationship between true positive rate (TPR) and false positive rate (FPR). For the 1 × 1 CV, the optimal threshold for classification (based on the MATLAB perfcurve function) gives 0.8286 TPR and 0.4714 FPR. This means that the classification procedure identified around 83% of Atypical MDD patients correctly, while still managing to classify healthy controls correctly around 53% of the time. It should be noted that the algorithm used for selecting the optimal classification threshold prioritizes TPR over FPR, as a false positive is assumed to be less harmful than a false negative.

## Discussion

### There is an unmet need for objective diagnosis

Major Depressive Disorder (MDD) is a mental health condition characterized by weight loss or gain, hypersomnia or insomnia, anhedonia, and persistent low mood among other symptoms (Belmaker and Agam, 2008). The MDD subtypes are characterized by varied symptoms: Melancholic (weight loss and insomnia), Atypical (weight gain and hypersomnia), and patients that are neither Melancholic nor Atypical (Gili et al., 2012).

Currently, there are no reliable objective tools available for diagnosing major depressive disorder (MDD) or guiding treatment for those suffering from this condition. Diagnosis primarily relies on clinical interviews with questionnaires based on the DSM-5 (Diagnostic and Statistical Manual of Mental Disorders, Fifth Edition) (American Psychiatric Association, 2013). However, even experienced interviewers face challenges in making accurate diagnoses, and significant variability exists among clinicians assessing the same patient (Regier et al., 2013). Factors affecting the reliability of diagnoses include comorbidities (Regier et al., 2013) and cultural influences on how symptoms are presented and reported (Sun et al., 2019).

Objective diagnosis based on measurable biomarkers would help address this challenge. For example, the hypothalamic-pituitary-adrenal (HPA) axis and the subsequent hormone regulation are reported to be dysregulated in patients with MDD (Yehuda et al., 1996; Gillespie and Nemeroff, 2005; Shea et al., 2005; Carroll et al., 2007; Fink and Taylor, 2007; Murri et al., 2014), though there is some dissent about what causes the dysregulation (Holsboer, 2000; Ceruso et al., 2020). Unfortunately, after much effort in classification of disease state based on TSST data, this goal remains elusive.

### The limitations of the current results

Given the small number of patient samples used in this work (reported tests were between healthy control and Atypical MDD subjects and used 29 individuals), we cannot be certain of the generalizability of this classification method. While the AUROC measure after thorough cross-validation procedures can give a degree of confidence, we cannot be fully certain that this will be replicable across datasets.

Further, the lack of explainable parameters in ML/AI methods gives us pause when assessing the reasonability of model decisions. By contrast, traditional regression models or mechanistic ODE models have parameters that are explicitly defined and comparable among the model parameters. While differences in the observed data can be used for classification purposes, we cannot explicitly determine which features of the data are used in decision making due to the largely black-box nature of neural networks. In the next section, we will address these concerns and provide an overview of how confidence in the results can be increased in the future.

A limitation of the method unrelated to the mathematical underpinnings of the classification is that conducting a TSST is expensive and stressful. This limits the applicability of the method in its current state, although we do not believe these limitations are insurmountable. For instance, the use of Virtual Reality TSST has been shown to elicit comparable stress reactions to live TSST (Shiban et al., 2016). This could significantly decrease the cost of obtaining additional data and any eventual clinical applications of the method.

### Perspective: how to build a stronger tool

There is much room for improvement in the data available for training. Ideally, further data from a larger population of control and MDD subjects, as well as other types of data, should be assembled for classification purposes. For example, positron emission tomography (PET) or functional magnetic resonance imaging (fMRI) scans, quantitative data on behavior changes, and other types of physiological indicators of stress could be used in combination with cortisol and ACTH. With the power of NODEs in translating time-series data into images ready for CNN classification, mixed types of data that include both time-series data and images from the same patients could be used together to maximize diagnostic accuracy.

The second concern we would address is with the lack of explainability in some ML/AI methods. There are interesting techniques, such as Layer-wise Relevance Propagation (LRP) (Bach et al., 2015), for explaining classification of images with CNNs. This method creates a heatmap of how relevant each pixel is to the final classification. The method has been extended to apply to recurrent neural networks (such as long short-term memory and gated recurrent unit networks) (Arras et al., 2017). The incorporation of such methods promises to make the results more explainable.

Using NODEs to learn the underlying vector field of a system is a first step towards a more complete understanding of the HPA axis under stress and how the system is dysregulated in MDD. With data scale and variety being overcome, objective diagnosis based on measurable biomarkers promises to aid clinicians in better helping MDD patients.

## Methods

### Data collection and selection

In clinical settings, there are two main avenues for physicians/researchers to investigate patient HPA axis dynamics. The first common test for diagnosis of HPA axis dysregulation is the Dexamethasone Suppression Test (DST). In a DST, a small dose (0.25–1.0 mg) of the synthetic glucocorticoid dexamethasone is administered in the evening and plasma cortisol is measured several times the next day (Gillespie and Nemeroff, 2005). While there have been interesting results regarding the changes in MDD patient responses to DST, we have chosen to focus on the second method: stress tests.

A Trier Social Stress Test (TSST) involves placing research subjects in a stressful situation (mock interview and surprise mental arithmetic test) while taking measurements regarding their stress response (Kirschbaum et al., 1993; Allen et al., 2017). The TSST data we have used was from repeated blood draws at 10 or 15-min intervals during the test—the blood was subsequently assayed for plasma ACTH and cortisol concentrations. This data allows for examination of the system dynamics on a much shorter and more granular timescale than a DST, including the initial uptick in ACTH/cortisol on exposure to a stressor and its subsequent decline to baseline due to feedback effects. We have used data from a TSST study to perform this research (see Parker et al. (2022) for details of data collection).

The number of control subjects was 15, and the total number of MDD subjects was 43 (roughly evenly distributed between the three subtypes). In Supplementary Figures S1, S2, we have carried out boxplots of the hormone area under the curve (AUC) measures in all these four groups.

In this work, we chose to carry out the classification between the healthy control subjects and Atypical MDD subjects so that the two groups being classified are roughly equal in numbers. The code provided in the Supplementary Material can be modified to carry out similar classification between healthy controls and Melancholic or Neither MDD subtype groups, further demonstrating the generality of our toolset.

### Artificial neural network (ANN)

In their most basic form, ANNs consist of a single fully-connected layer to perform a linear combination of the inputs followed by a non-linear activation function (such as ReLU or hyperbolic tangent) (Aggarwal, 2018). Adding in additional fully-connected layers (or additional hidden nodes in each layer) increases the complexity of the model and is thereby expected to improve performance to an arbitrarily accurate level given enough data (by the Universal Approximation Theorem (Hornik et al., 1989; Yu, 2021)).

In practice, however, this is not the case when using small datasets. Without sufficient data, increasing the size of any NN increases overfitting of the training data and thereby decreases generalizability (Aggarwal, 2018). Further, ANNs do not allow us to consider the data as a time-series, which loses information contained in the data. Therefore, we have turned to several network architectures that can accept time-series data as inputs.

### Neural ordinary differential equation (NODE)

Another network architecture that we have applied is NODE—first introduced by Chen et al. (2018). NODEs represent the continuous time extension of Residual Neural Networks (ResNets). The architecture of a ResNet is a network containing residual connections in some subnetworks. These residual connections lead to the relation between inputs and outputs seen in Eq. 1.

$$y=F\left(x\right)+x$$ (1)

where x represents the vector of inputs, y represents the vector of outputs, and F is a function representing the operations performed by the residual subnetwork. The residual connection passes the inputs directly through to sum with the outputs—propagating the input signal deeper into the network in part to combat the vanishing gradient problem (Aggarwal, 2018).

In deep ResNets, multiple residual subnetworks (often called Residual Blocks) are stacked. If we imagine stacking an infinite number of Residual Blocks, with each taking the inputs, adding some value depending on the inputs and then passing it to the next block, this is approximately equivalent to the NODE architecture (Dupont et al., 2019). In NODEs, the system has its right-hand side represented by a NN as shown in Eq. 2.

$$\frac{dz}{dt}=f_{NN}\left(z,t,\theta \right)=f_{\theta }\left(z\right)$$ (2)

Where z is the vector of variable states and θ represents the matrix of parameters (weights and biases) of the network. This system is residual because each time step of integration by the differential equation solver increments the output from the previous step. Unlike discrete-time ResNets, this process uses an adaptive step size to take an arbitrary number of steps covering the desired time interval of integration. It should be noted that Massaroli et al. (2020) dispute the equivalence of NODEs and infinite ResNets due to the depth-invariance of the parameters in the original NODE formulation. Every time step of the system described above has the same parameters θ applied, while ResNets have a new parameter matrix for every layer.

### Network training

We performed the network training with the AdamW optimizer using 1e-6 weight decay, 1e-3 learning rate and mean squared error (MSE) loss. Activations were ReLU by default, although we tested using alternatives including hyperbolic tangent. All code was written in Python using PyTorch and torchdiffeq packages for NN operations. All networks were trained/tested on a 2020 Macbook Pro with an Apple M1 chip. See Supplementary Material S1 for the full code archive. Various figures were generated in MATLAB (using the results from the Python code) to make use of the GUI for customization.

## Data Availability

Publicly available datasets were analyzed in this study. This data can be found here: CP, EN, TZ. VeVaPy, a Python Platform for Efficient Verification and Validation of Systems Biology Models with Demonstrations Using Hypothalamic-Pituitary-Adrenal Axis Models. Entropy. 2022; 24(12):1747. https://doi.org/10.3390/e24121747.
