# Supplementary figures and images for: Applying neural ordinary differential equations for analysis of hormone dynamics in Trier Social Stress Tests
Source: Front Genet. 2024 Aug 1;15:1375468. doi: 10.3389/fgene.2024.1375468 (PMC11324453; doi:10.3389/fgene.2024.1375468)

# Figure S1: ACTH AUC Boxplots By Patient Group

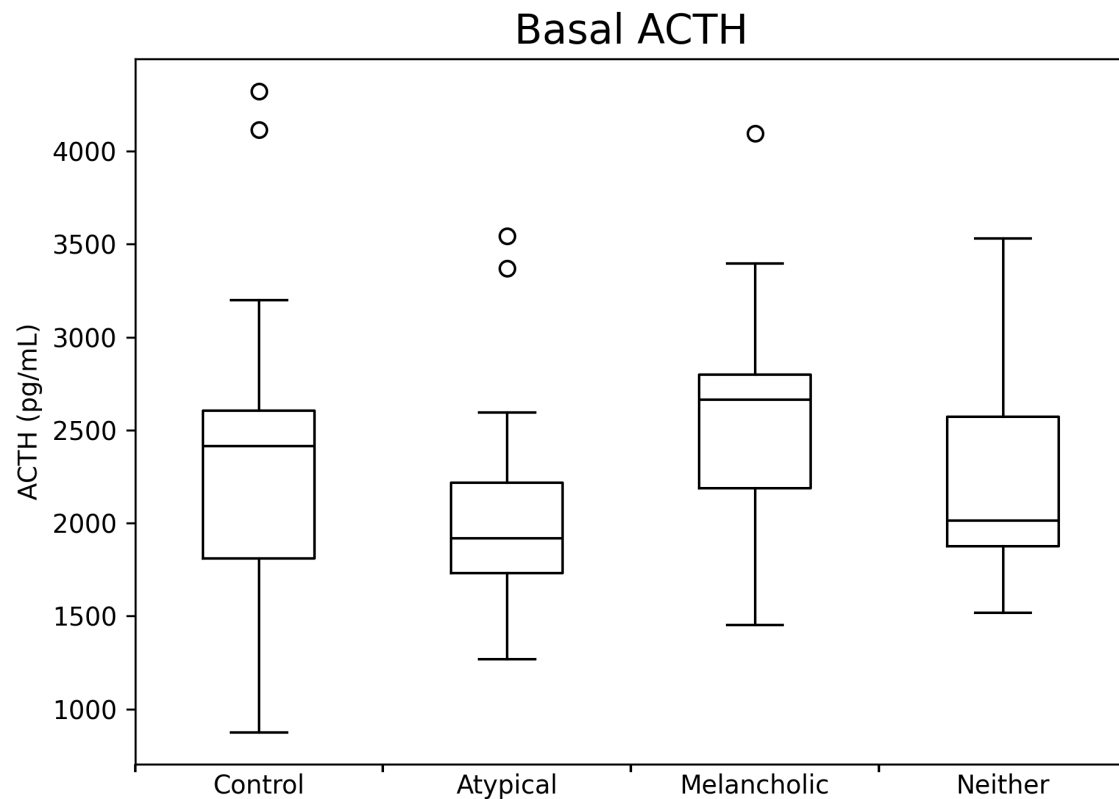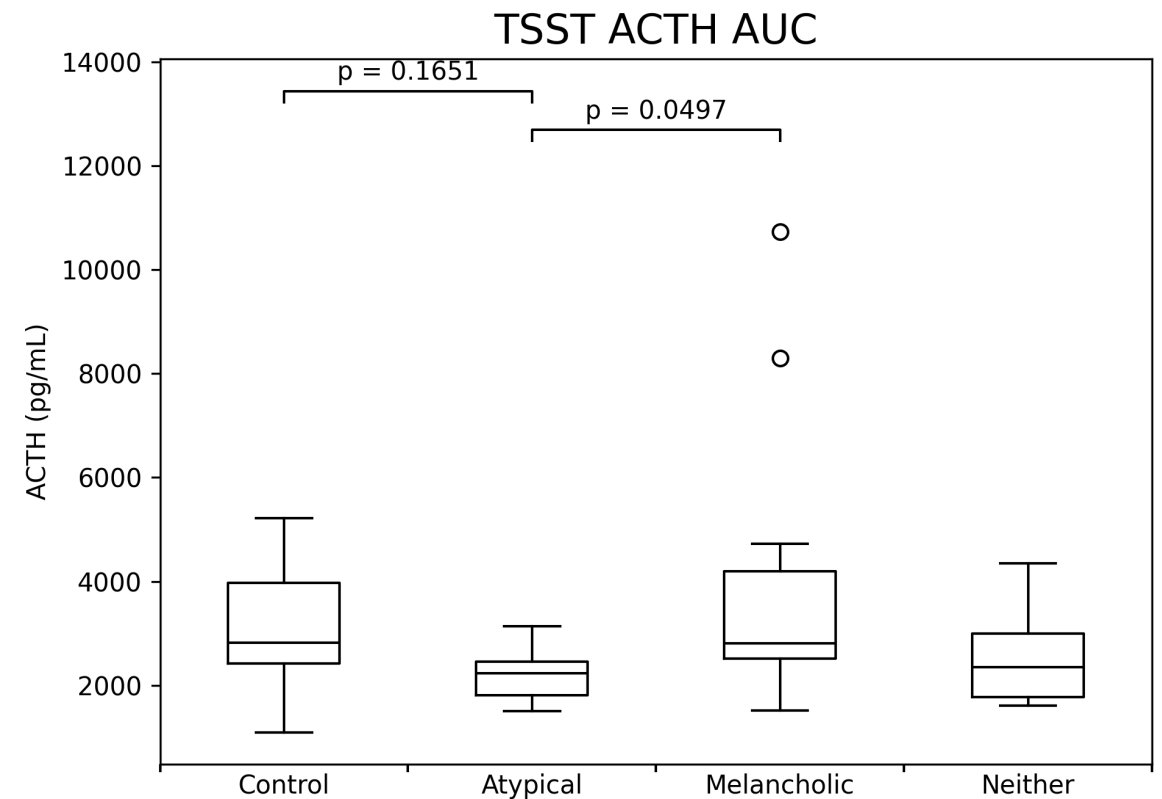

# Figure S2: Cortisol AUC Boxplots By Patient Group

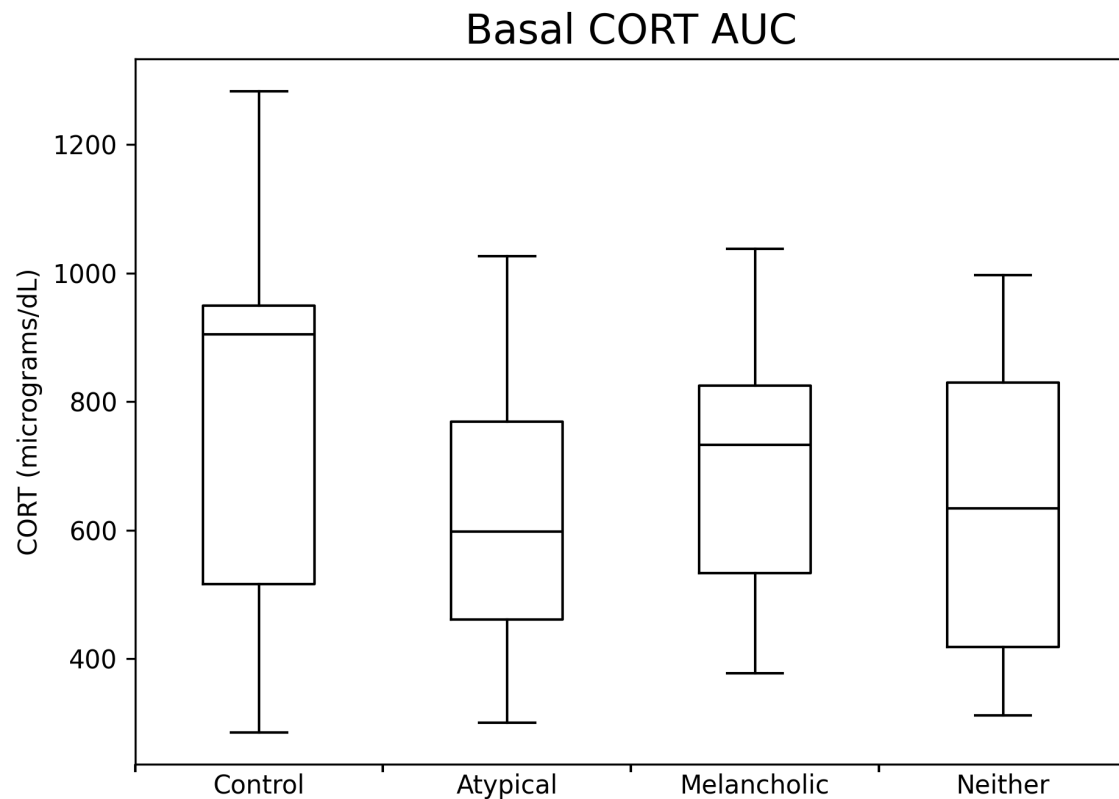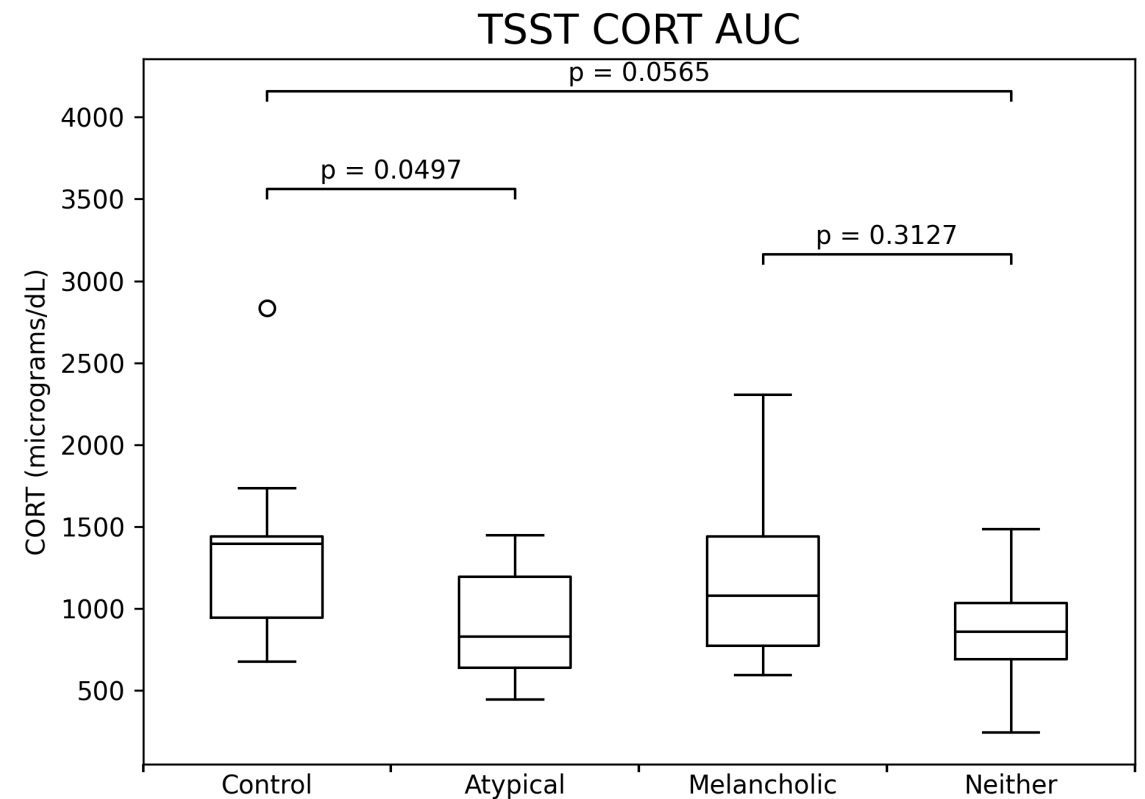

Supplement: Supplementary file 1 [file DataSheet1.PDF]
